# Special Issue: “Unraveling the Involvement of Adipose Tissue in Breast Cancer Progression”

**DOI:** 10.3390/ijms22105107

**Published:** 2021-05-12

**Authors:** Tiziana Triulzi

**Affiliations:** Molecular Targeting Unit, Department of Research, Fondazione IRCCS Istituto Nazionale dei Tumori, 20133 Milan, Italy; tiziana.triulzi@istitutotumori.mi.it; Tel.: +39-(02)-2390-5121

White adipose tissue (WAT) is a heterogeneous tissue that is composed of adipocytes and several non-adipocyte cell populations, including adipose progenitors, fibroblasts, endothelial and infiltrating immune cells. This Special Issue includes eight papers, four reviews and four original articles that explore the cellular and molecular players in the detrimental cross-talk between adipose tissue and breast cancer (BC). Studying this cross-talk could pave the way toward novel strategies for BC treatment, not just for obesity.

BC has long been accepted to be an obesity-associated cancer. Indeed, strong epidemiological evidence links obesity with the incidence of BC and a poor prognosis. Experimental studies in vitro and in vivo with genetically modified and diet-induced obese (DIO) animals suggest a complex role of obesity in the initiation and progression of BC (reviewed in [1]); however, the underlying cellular and molecular factors are not completely understood. All components of WAT—not only adipocytes—that are interconnected in a network of abnormal regulation and intercellular signaling, govern the development of obesity, regulating local and systemic alterations in obesity-related WAT (e.g., inflammation, altered metabolism, fibrosis, oxidative stress) that negatively impact cancer development and progression [1]. Normalization of these disrupted interactions is a potential target of future preventive and therapeutic strategies to overcome obesity-associated drug resistance and improve patient prognosis. In this context, aspirin has been tested in vitro with regard to the communication between 3T3-L1 inflamed adipocytes and 4T1 BC cells, to hamper obesity-related inflammation [2]. Reduced inflammation and attenuation of obesity-related metabolism and pro-inflammatory fatty acids were observed in adipocytes, concomitant with the inhibition of obesity-associated cancer cell growth and migration [2].

Possible support in tumor progression is also provided by normal adipose tissue. Using a new 3D culture model, Asante et al., demonstrated that murine subcutaneous WAT from lean and obese mice similarly induces an aggressive hybrid epithelial/mesenchymal phenotype in MDA-MB-231 triple-negative (TN) mesenchymal cells [3], suggesting that also under normal-weight conditions, the interaction between tumor cells and adipose tissue fosters tumor progression. Recently, even in normal-weight conditions, it has been shown that the direct cross-talk between BC cells and adipocytes leads to the generation of cancer-associated adipocytes (CAAs), and the acquisition of an aggressive cancer phenotype (reviewed in [4]). Few studies have identified molecules (Wnt3A, Wnt5A, IL6, adrenomedullin, TGFβ1 and Notch1) that are implicated in the induction of adipocyte dedifferentiation, delipidation, and the acquisition of an activated phenotype that is typical of CAAs in cancer and other pathologies [4]. Because these molecules are known to be part of the complex adipogenesis program as inhibitors [4], it is likely that a deep understanding of the regulation of adipogenesis signaling pathways will provide clues for cancer therapies. Analogous to features of dysfunctional, obesity-associated adipocytes, molecules and metabolites that are relevant in CAA-driven cancer progression have been proposed [4]. Determining the functions and mechanisms of these molecules in BC progression is the next step in establishing CAA-targeted therapies as a new strategy to slow tumor progression.

One such example is autotaxin (ATX), the expression of which increases in adipose tissue primarily in obesity and on interaction with tumor cells. Through the production of lysophosphatidate (LPA), ATX controls a wide range of signaling pathways in wound healing, chronic inflammation, cancer progression and resistance to cancer therapy (reviewed in [5]). ATX and LPA inhibitors have been proven to be safe in clinical studies for pulmonary fibrosis, and thus could be easily repurposed as adjuvant treatments for cancer to block the ATX-LPA-inflammation cycle [5].

Another possible target in adipocyte-tumor cell cross-talk is lactate dehydrogenase (LDH), a key metabolic enzyme in the conversion between pyruvate and lactate that regulates nutrient exchange between tumor and stromal cells in the tumor microenvironment, supporting tumor progression. Higher lactate production, concomitant with higher LDHA (converting pyruvate to lactate) and lower LDHB (converting lactate to pyruvate) expression in malignant BC compared with benign disease, has been observed, mainly in normal-weight patients [6]. Adipose tissue that is adjacent to tumor tissue increases LDH activity—primarily LDHB—facilitating lactate oxidation in adipocytes in stromal–epithelial metabolic coupling [6].

The ’unhealthy’ phenotype of CAAs and cells in the adipose tissue of obese patients and their pro-tumoral activity leads to question about lipofilling, procedure, which is used widely in breast reconstructive surgery, primarily after BC surgery. Whereas clinical data support the safety of this procedure, showing that it does not increase the risk of BC reoccurrence, pre-clinical in vitro studies have reported contradictory results, with some studies demonstrating the pro-tumorigenic potential of adipocytes and adipose-derived stromal cells of the lipoaspirate (reviewed in [7]). Similarly, contradictory results have been generated by Tokumaru et al., who analyzed the clinical relevance of the amount of intratumoral mature adipocytes, defined transcriptionally through xCell deconvolution [8]. Whereas adipocyte-high tumors were significantly enriched for inflammation and metastasis-related gene sets, consistent with in vitro data, they were also associated with a favorable tumor immune microenvironment, low proliferation and a low grade only in ER+/HER2- tumors [8]. These data were not confirmed in TNBCs or HER2+ tumors, despite adipocyte levels having no prognostic or predictive value with regard to chemotherapy resistance in all intrinsic subsets [8]. These data implicate differences in adipocyte-tumor cell cross-talk, according to tumor-intrinsic features, and encourage further studies to measure the prognostic impact of adipocytes that infiltrate BC tissues and their phenotype under normal-weight conditions. Such an analysis would also be instrumental in determining whether they can be targeted as a new therapeutic strategy in cancer patients.
